# Click-Derived Triazoles and Triazolylidenes of Manganese for Electrocatalytic Reduction of CO_2_ †

**DOI:** 10.3390/molecules26216325

**Published:** 2021-10-20

**Authors:** Sofia Friães, Sara Realista, Clara S. B. Gomes, Paulo N. Martinho, Beatriz Royo

**Affiliations:** 1 ITQB NOVA, Instituto de Tecnologia Química e Biológica António Xavier, Av. da República, 2780-157 Oeiras, Portugal; sofiafriaes@itqb.unl.pt (S.F.); sara.realista@itqb.unl.pt (S.R.); 2 LAQV-REQUIMTE, Department of Chemistry, Campus de Caparica, NOVA School of Science and Technology, NOVA University Lisbon, 2829-516 Caparica, Portugal; clara.gomes@fct.unl.pt; 3 Associated Laboratory i4HB-Institute for Health and Bioeconomy, School of Science and Technology, NOVA University Lisbon, 2829-516 Caparica, Portugal; 4 UCIBIO-Applied Molecular Biosciences Unit, Department of Chemistry, School of Science and Technology, NOVA University Lisbon, 2829-516 Caparica, Portugal; 5 Biosystems and Integrative Sciences Institute (BioISI), Faculdade de Ciências, Campo Grande, Universidade de Lisboa, 1749-016 Lisboa, Portugal; pnmartinho@fc.ul.pt; 6 Centro de Química Estrutural, Campo Grande, Faculdade de Ciências Universidade de Lisboa, 1749-016 Lisboa, Portugal

**Keywords:** manganese, mesoionic carbenes, electrocatalytic CO_2_ reduction

## Abstract

A series of new *fac*-[Mn(L)(CO)_3_Br] complexes where L is a bidentate chelating ligand containing mixed mesoionic triazolylidene-pyridine (MIC^py, **1**), triazolylidene-triazole (MIC^trz, **2**), and triazole-pyridine (trz^py, **3**) ligands have been prepared and fully characterized, including the single crystal X-ray diffraction studies of **1** and **2**. The abilities of **1**–**3** and complex *fac*-[Mn(MIC^MIC)(CO)_3_Br] (**4**) to catalyze the electroreduction of CO_2_ has been assessed for the first time. It was found that all complexes displayed a current increase under CO_2_ atmosphere, being **3** and **4** the most active complexes. Complex **3**, bearing a N^N-based ligand exhibited a good efficiency and an excellent selectivity for reducing CO_2_ to CO in the presence of 1.0 M of water, at low overpotential. Interestingly, complex **4** containing the strongly electron donating di-imidazolylidene ligand exhibited comparable activity to **3**, when the experiments were performed in neat acetonitrile at slightly higher overpotential (−1.86 vs. −2.14 V).

## 1. Introduction

**Scheme 1:**
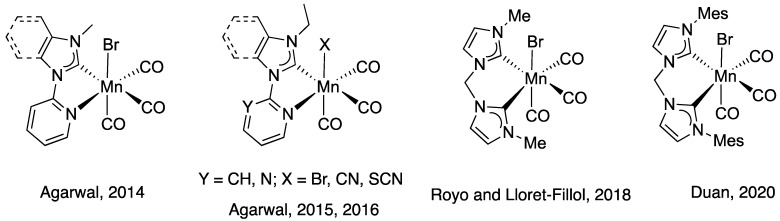
Mn(I)-NHC complexes applied as electrocatalysts for the reduction of CO_2_ previously described in the literature.

Nowadays, the synthesis of renewable fuels from carbon dioxide (CO_2_) is a key strategy to solve the problems of global warming and fossil fuel shortages [1]. Among the available methods for CO_2_ fixation, the electrocatalytic reduction of CO_2_ represents a promising approach for the production of value-added chemicals as fuels [2,3]. In the last decade, intensive research has been done on CO_2_ electrocatalytic reduction using molecular catalysts in the last decade [4]. Initially, most of the studies focused on complexes with noble metals (Pd, Ru, Re) [4]. However, due to the need of replacing expensive metals by Earth-abundant cheap metals, the focus recently shifted to 3d metal such as Mn, Fe, Co, and Ni [5,6,7,8,9]. In particular, molecular Mn catalysts have proved to be very competitive for CO_2_ reduction [10,11]. In 2011, Deronzier and co-workers demonstrated for the first time the electrochemical reduction of CO_2_ mediated by *fac*-[Mn(bpy)(CO)_3_Br] (bpy = 2,2-bipyridine) complexes [12]. Later, several groups have investigated in detail the electrocatalytic activity of related pyridine-based Mn complexes [13,14,15,16,17]. The replacement of the bipyridyl ligand by mixed pyridyl-*N*-heterocyclic carbene ligands (NHC) has been explored by the Agarwal group (Scheme 1). Complexes of the general type *fac*-[Mn(py-NHC)(CO)_3_X] (NHC = imidazole- and benzimidazole-NHCs) resulted to be catalytically active for CO_2_ reduction and exhibited a two-electron reduction at a single potential rather than two single-reductions at separate potentials [18,19,20].

Recently, our group described the unique reactivity of *fac*-[Mn(bis-NHC^Me^)(CO)_3_Br] (Scheme 1) for the selective electrocatalytic reduction of CO_2_ to CO, showing the highest TOF_max_ value (ca. 320,000 s^−1^) ever reported for a Mn-based catalyst [21]. Interestingly, for the first time, the Mn tetracarbonyl intermediate [Mn(CO)_4_(bis-NHC^Me^)]^+^, was detected by IR under catalytic conditions. Following our work, similar studies were later performed by Duan and co-workers with a related Mn-NHC compound bearing the mesityl N-wingtip substituents instead of the methyl group (Scheme 1) [22].

In this work, keeping up with our interest in Mn-NHC complexes for catalysis [23,24,25,26,27] and in particular for CO_2_ reduction [21], we have developed a new family of Mn(I) tricarbonyl complexes with chelating ligands containing combinations of the mesoionic triazolylidene (MIC), triazole, and pyridine ligands and explored their activity in the CO_2_-electrocatalytic reduction. We became interested in exploring the impact of the presence of triazolylidene, triazole, and pyridine fragments in the coordination sphere of Mn. While the pyridine ring π-system is typically considered electron-deficient, triazolylidenes, an interesting subclass of NHC ligands, are strongly σ-donating ligands [28]. Hence, the introduction of the mesoionic triazolylidene ligands leads to an increase in the overall donor capacity of the ligands, being the triazole unit the poorest donor ligand of the triazolylidene/pyridine/triazole series [29,30]. The change in the electronic nature of the bidentate ligands may have important implications in the catalytic activity of their metal complexes. Recent advances in triazolylidene chemistry of first-row transition metals have shown the great potential of this type of ligands in catalysis [31,32,33,34]. Surprisingly, catalytic systems based on Mn-triazolylidene are limited to one example recently reported by us [26].

## 2. Results and Discussion

### 2.1. Synthesis and Characterization of Mn Complexes

**Scheme 2:**
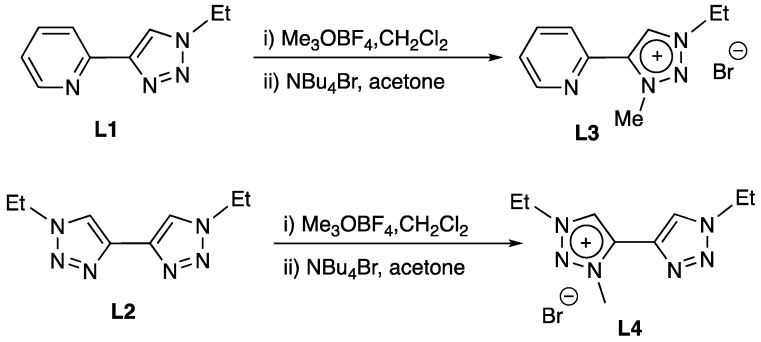
Preparation of the triazolium-derived bromide salts **L3** and **L4**.

The synthesis of ligands **L1**, **L2** was performed following the well-established copper-catalyzed click [3 + 2] cycloaddition reaction procedures that has been previously reported in the literature [35,36]. The triazolium-derived bromide salts **L3** and **L4** were prepared by methylation of the corresponding ligand precursors **L1** and **L2**, respectively, with trimethyloxonium tetrafluoroborate (Me_3_OBF_4_) [37,38], followed by anion exchange with tetra-n-butylammonium bromide (TBAB) (Scheme 2). **L3** and **L4** were characterized by NMR spectroscopy (Figures S1–S4).

**Scheme 3:**
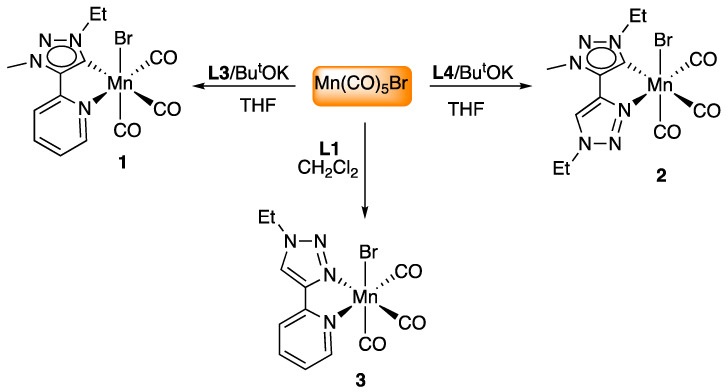
Synthesis of manganese Complexes **1**–**3**.

The new manganese complexes **1** and **2** containing a triazolylidene fragment were conveniently prepared by reaction of the appropriate ligands **L3** and **L4**, respectively, with [MnBr(CO)_5_] in the presence of one equivalent of ^t^BuOK (Scheme 3). Both complexes **1** and **2** were isolated as yellow crystalline solids in good yields (58% and 46%, respectively), and were fully characterized by NMR and IR spectroscopy, elemental analysis, and by single crystal X-ray diffraction studies (Figures S5–S13). Coordination of ligands **L3** and **L4** was confirmed by the disappearance of the signal of the triazolium proton at 9.61 and 9.36 ppm, respectively, in the ^1^H NMR spectra, and by the appearance of the characteristic resonance of the metalated carbon at 188.65 ppm (for **1**) and 185.83 (for **2**), in the ^13^C NMR spectra.

Complex **3** was easily prepared by direct reaction of [MnBr(CO)_5_] with the di-triazole ligand **L1** (Scheme 3) and it was characterized by NMR and IR spectroscopy, and elemental analysis. The metalation of **L1** was corroborated by a downfield shift of the triazole proton in the ^1^H NMR spectra and by the appearance of the characteristic resonances of CO ligands at 220.82–222.92 ppm in the ^13^C NMR.

The carbonyl ligands, in all complexes, showed the expected pattern for *fac*-tricarbonyl ligands in the IR spectra. The symmetrical CO stretching vibrations of Complex **3** appears at higher wavenumbers than those observed in complexes **1** and **2**, reflecting the weaker donor capacity of triazole moiety compared to the triazolylidene fragment (Table 1).

**Figure 1:**
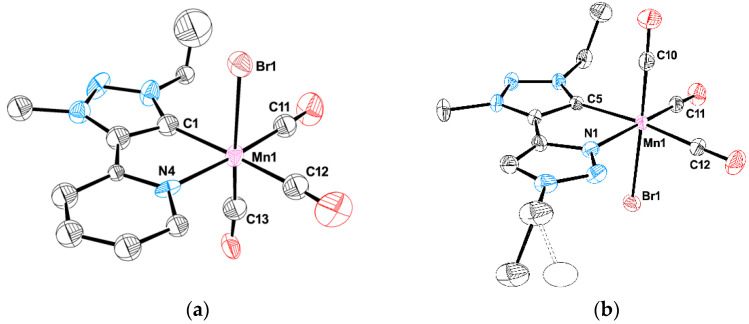
ORTEP-3 diagrams of (**a**) Complex **1** (molecule 1), and (**b**) Complex **2**, using 30% probability ellipsoids. All hydrogen atoms were omitted for clarity. In **2**, the carbon atom C4 is disordered over two positions with 49 and 51% probability, respectively, PART B being depicted as a dashed bond and sphere. Selected bond lengths for **1**: Mn1–N4 2.15(3) Å, Mn1–C1 1.96(3) Å, Mn1–Br1 2.525(6) Å, Mn1–C11 1.73(3) Å, Mn1–C12 1.84(3) Å, Mn1–C13 1.66(3) Å. Selected bond lengths for **2**: Mn1–C5 2.031(4) Å, Mn1–N1 2.065(3) Å, Mn1–Br1 2.5554(7) Å, Mn1–C10 1.781(5) Å, Mn1–C11 1.782(4) Å, Mn1–C12 1.831(4) Å.

The molecular structures of complexes **1** and **2** were established by X-ray diffraction analyses. Figure 1 shows their ORTEP-3 diagrams, with the most relevant bond distances reported in the corresponding caption. Both structures reveal a coordination geometry around the Mn(I) center as slightly distorted octahedral with three facially disposed CO ligands. The Mn–C and Mn–N bond lengths (Figure 1, caption) are comparable to values observed in previously reported complexes [26,27].

### 2.2. Electrocatalytic Reduction of CO_2_ Mediated by Mn Complexes ***1**–**4***

#### 2.2.1. CV Studies Performed under Nitrogen Atmosphere

**Scheme 4:**
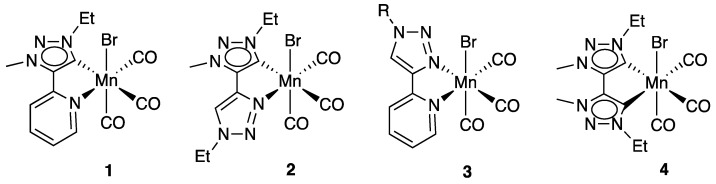
Mn complexes **1**–**4** studied in CO_2_ electrocatalytic reduction.

We decided to explore the catalytic activity of complexes **1**–**4** (Scheme 4) in the electrocatalytic reduction of CO_2_. First, cyclic voltammetry (CV) was used to investigate the electrochemical reduction of the new complexes **1**–**3** under nitrogen atmosphere in acetonitrile solutions (Figure S14). All potentials were referred to the Fc^+/0^ couple. The synthesis and CV studies of complex **4** has been already reported by us [27].

Complex **1** showed three reduction processes at −1.85, −2.19 and −2.75 V. These reduction events were in line with those found for the manganese di-triazolylidene parent compound **4**, previously reported by us [27]. Interestingly, the exchange of one triazolylidene moiety in **4** by a pyridine unit led to an anodic shift of the first and second reduction peaks of more than 250 mV. When the pyridine unit in **1** was replaced by a triazole fragment giving complex **2**, only two reduction processes were observed, and these two reduction events were apart for more than 500 mV. As expected, complex **3** showed a similar reduction behavior than that observed for other Mn complexes containing N-based ligands of the general formula [Mn(N^N)(CO)_3_Br] [16], which indicated that the first reduction is likely to comprise a 2e^−^ reduction process. The presence of the pyridine unit in **3** in place of the triazole fragment has a direct impact in the reduction potential.

Although the redox potentials obtained from the cyclic voltammetry studies are often used to evaluate the donor/acceptor properties of the ligands in metal complexes, the IR stretching bands in **1**–**3** did not correlate with the observed trend in their redox potentials. Relationship between redox potentials and ligand donor ability is not straightforward as redox potentials are global probes. These correlations are more accurate when the oxidation is metal-based and the reductions are ligand-based. For complexes **1**–**4** the reduction is most likely metal-based, what would explain the lack of correlation between their IR and CV [29].

#### 2.2.2. CV Studies Performed under CO_2_ Atmosphere

**Figure 2:**
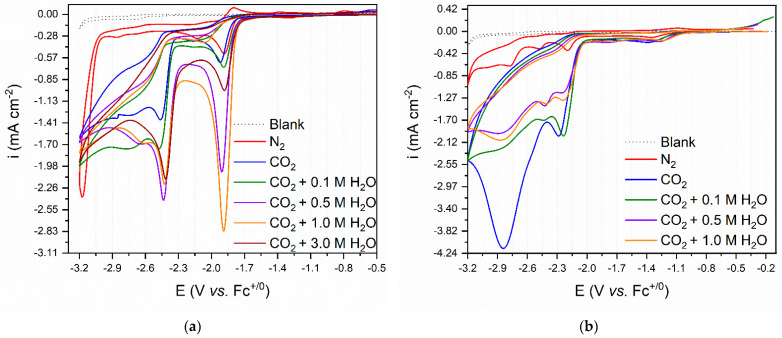
Cyclic voltammograms of Complex **3** (**a**) and **4** (**b**) (1 mM) in MeCN, N_2_ or CO_2_ saturated solutions using TBAPF_6_ as supporting electrolyte (0.1 M) at 0.1 V s^−1^ in the presence of different [H_2_O]. Glassy carbon (3 mm diameter) was used as working, platinum wire as counter and Ag wire as pseudo-reference electrodes.

Next, complexes **1**–**4** (1 mM in acetonitrile solutions) were studied as catalysts precursors for the electroreduction of CO_2_. Under CO_2_ atmosphere, and without the addition of a proton source, it was observed a current enhancement for all complexes (Figure 2 and Figures S15–S16). The highest i_cat_/i_p_ ratio was obtained for **4**, followed by **3** (Figure S17).

When these experiments were performed in the presence of water (0.1–3.0 M) as a proton source, different effects depending on the catalyst were observed. In the case of **3**, a new reduction process is observed at −2.43 V under CO_2_ atmosphere, along with the reduction processes already observed under N_2_ (around −1.90 V), Figure 2. Interestingly, for **3**, the presence of >0.1 M of H_2_O promoted a stronger increase of the catalytic current at lower potentials (−1.84 V) than that observed at higher potentials (−2.42 V). This trend was not observed in complexes **1**, **2** and **4**. When complexes **1** and **2** were used under similar conditions (in the presence of >0.1 M of H_2_O), the presence of water did not produce a significant catalytic current increase (Figures S15–S17). In comparison, when the experiments were performed using **4**, the current immediately decreased in the presence of H_2_O, suggesting low stability of **4** under these conditions.

Table 2 shows the values of the E_cat/2_ and (i_cat_/i_p_)^2^ for **1**–**4**, using for each case an optimized concentration of H_2_O that was measured by the highest catalytic current observed. It is possible to conclude that complexes bearing the triazolylidene unit **1**, **2** and **4** are less tolerant to the addition of water than complex **3**, and for them, the highest catalytic current was observed when 0.1 M of water is present. In fact, for **4** the catalytic current decreased right after the lowest concentration of water was added (0.1 M). This result indicates that for **1**, **2** and **4**, the catalytic system becomes unstable, leading to the degradation of the complexes, and their deposition on the electrode surface [22]. In particular, when **4** is used in the presence of 0.1 M of water, the successive current increases between cycles indicates deposition of electroactive material on the electrode. This was confirmed in a bulk electrolysis experiment where a white deposit was observed on the working electrode surface.

As shown in Table 2, the electrocatalytic activity of **1** and **2** was lower than that of **3** and **4**, as observed by their lower (i_cat_/i_p_)^2^ values (<5) compared to those of **3** and **4** (>20). Interestingly, complex **3** displayed a low catalytic potential in the presence of water (−1.86 V).

Considering the beneficial effect of the addition of water in the catalytic performance of complex **3**, we decide to explore the effect on the addition of a different proton source. Thus, the addition of 2,2,2-trifluoroethanol (TFE) was investigated. However, using TFE, no higher activity, as the highest (i_cat_/i_p_)^2^ value obtained was 9.80, and no significant difference on the E_cat/2_ was observed (Figure S18–S20).

#### 2.2.3. Bulk Electrolysis Experiments

Bulk electrolysis experiments were performed to quantify the CO_2_ reduction products obtained in the electrocatalytic reduction of CO_2_ mediated by **3** and **4**, which were the complexes that displayed the best catalytic performances**.** The experiments were carried out using the optimal conditions determined by the CV studies. Therefore, bulk electrolysis with **4** was performed at two different potentials (−2.08 V and −2.15 V) in acetonitrile and in the absence of water, while bulk electrolysis using **3** was performed with and without H_2_O or TFE as proton sources applying the same potential (ca. −1.9 V). The gaseous headspace of the bulk electrolysis was analyzed by gas chromatography with thermal conductive detection (GC-TCD), and the liquid phase by high performance liquid chromatography (HPLC) with UV-vis and refraction index detections.

In Table 3 are summarized the data for the bulk electrolysis experiments, and the current-time and charge-time graphs are provided in Figures S21–S22. The electrolysis potential was chosen by observing the cyclic voltammograms and recorded at the middle of the catalytic wave. Under the conditions indicated above, in neat acetonitrile, compound **4** was studied at two different potentials (−2.08 and −2.15 V), being the lowest one the most favorable, observed by the most stable current versus time behavior (Figure S22). At an applied potential of −2.08 V, complex **4** led to catalytic production of CO with a faradaic efficiency (FE_CO_) of 70%, without any detectable traces of H_2_. At the end of the bulk electrolysis experiments a TOF_CO_ value of 4.08 h^−1^ was obtained, that is lower than the one obtained for [Mn(bis-NHC^Me^)(CO)_3_Br] catalyst (14 h^−1^, calculated after bulk electrolysis experiments using TON = n_CO_/n_cat_ and TOF = TON/time), previously reported by us [5,21].

In the case of complex **3**, when the experiments were performed in the absence of a protic source, a strong decrease of the current versus time during the bulk electrolysis was observed, and a production of CO with a FE_CO_ value of 56% was obtained (Table 3, entry 1, and Figure S21). The decrease in the current is probably due to the fast deactivation of the catalyst under these conditions. When the experiments using **3** are performed in the presence of water or TFE, the deactivation of **3** is mitigated, and at an applied potential of −1.85 V, the production of CO increased to a FE_CO_ value of 72% (when 0.5 M of water H_2_O is used) (Table 3, entry 2). The reaction was selective for the formation of CO; formation of H_2_ was not detected and only a negligible amount of formic acid (FE < 8%) was observed in the liquid phase. The FE_CO_ values obtained using **3** are comparable to those reported in the literature for other manganese tricarbonyl complexes bearing N-based ligands [5]. However, it is important to highlight that the potential used in the CO_2_ reduction mediated by **3** is significantly low compared to the majority of the Mn complexes reported in the literature, making **3** a promising energy-efficient catalyst for CO_2_ reduction [5]. A TOF_CO_ value of 3.67 h^−1^ calculated after bulk electrolysis experiments was obtained for **3** using 1.0 M of TFE at an applied potential of −1.92 V, which compares well with values reported for other Mn tricarbonyl complexes reported in the literature [5].

## 3. Materials and Methods

### 3.1. General Considerations

All reactions and manipulations were performed under a nitrogen atmosphere using standard Schlenk techniques. Solvents were purified from appropriated drying agents and distilled under nitrogen before use. All reagents were purchased from commercial suppliers and used without further purification. ^1^H and ^13^C NMR were recorded with Brucker Avance III 400 MHz. Elemental analyses were performed in the laboratories at ITQB. Ligands **L1** [35] and **L2** [36], and Mn Complex **4** were prepared according to previously described procedures [26].

### 3.2. Synthetic Procedures

#### 3.2.1. Preparation of Triazolium Bromide Salt **L3**

The triazolium bromide salt **L3** was prepared by counter-ion exchange from the corresponding triazolium tetrafluoroborate salt, which has been reported in the literature [38]. A mixture of **L1** (1 eq.) and *m*-chloroperoxybenzoic acid (2 eq.) was suspended in chloroform (15 mL) and refluxed for 1 h. After cooling to room temperature, the mixture was poured into CH_2_Cl_2_ (100 mL) and washed with aqueous KOH solution (1 M, 3 × 50 mL). The organic layer was separated, dried over Na_2_SO_4_ and the solvent evaporated to dryness to give a white solid, which was dissolved in dry dichloromethane (10 mL) and Me_3_OBF_4_ (4 eq.) was added. Then, the mixture was stirred for 4 days at room temperature. All volatiles were removed under vacuum, the remaining residue was suspended in dry ethanol (40 mL), Mo(CO)_6_ (1 eq.) was added, and the mixture refluxed for 1 h. After cooling to room temperature, the solvent was removed under vacuum, and the crude was purified by silica gel column chromatography using CHCl_3_/MeOH (10:1) as eluent to yield the corresponding triazolium tetrafluoroborate salt in a pure form. The triazolium tetrafluoroborate salt was then dissolved in a minimum amount of acetone, and tetrabutylammonium bromide (TBAB) (2 eq.) was added. The mixture was stirred at room temperature for 2 h, leading to the formation of a white precipitate, corresponding to **L3**, which was isolated by filtration and washed with acetone and ether.

**L3**. Yield: 205 mg (75%). ^1^H NMR (400 MHz, DMSO-d_6_) δ: 9.61 (s, 1H, CH_trz_), 8.85 (s, 1H, CH_py_), 8.15 (t, J = 7.77 Hz, 1H, CH_py_), 8.07 (d, J = 7.91 Hz, 1H, CH_py_), 7.68–7.65 (m, 1H, CH_py_), 4.71 (q, J = 7.34 Hz, 2H, NCH_2_CH_3_), 4.56 (s, 3H, NCH_3_), 1.61 (t, J = 7.34 Hz, 3H, NCH_2_CH_3_). ^13^C NMR (100 MHz, DMSO-d_6_) δ: 150.11 (CH_py_), 143.21, 140.12, 138.36 (CH_py_), 129.29 (CH_trz_), 125.81 (C_py_), 124.44 (C_trz_), 48.95 (NCH_2_CH_3_), 40.71 (NCH_3_), 13.92 (NCH_2_CH_3_).

#### 3.2.2. Preparation of Triazolium Bromide Salt **L4**

A mixture of **L2** (1 eq.), Me_3_OBF_4_ (1 eq.) and dry CH_2_Cl_2_ (10 mL) was stirred at room temperature for 3 days under nitrogen atmosphere. Then addition of n-hexane produced the precipitation of a white solid that was isolated by filtration. The obtained white solid was re-dissolved in a minimum amount of acetone, TBAB (2 eq.) was added, and the reaction mixture was stirred at room temperature for 2 h. The desired product **L4** precipitated from the reaction mixture as a white solid, which was isolated by filtration and washed with acetone and ether.

**L4**. Yield: 130 mg (54%). ^1^H NMR (400 MHz, DMSO-d_6_) δ: 9.36 (s, 1H, CH_trz_), 9.00 (s, 1H, CH_trz_), 4.69 (q, J = 7.22 Hz, 2H, NCH_2_CH_3_), 4.56 (q, J = 7.23 Hz, 2H, NCH_2_CH_3_), 4.46 (s, 3H, NCH_3_), 1.58 (t, J = 7.22 Hz, 3H, NCH_2_CH_3_), 1.52 (t, J = 7.22 Hz, 3H, NCH_2_CH_3_). ^13^C NMR (100 MHz, DMSO-d6, 298 K) δ: 134.40 (C_trz_), 131.46 (C_trz_), 127.62 (CH_trz_), 125.62 (CH_trz_), 48.95 (NCH_2_CH_3_), 45.46 (NCH_2_CH_3_), 15.23 (NCH_2_CH_3_), 14.03 (NCH_2_CH_3_).

#### 3.2.3. Preparation of Complexes **1** and **2**

Solid [MnBr(CO)_5_] (1.2 eq.) was dissolved in dry THF (20 mL) and ^t^BuOK (1.2 eq.) was added to the mixture, which was heated to 60 °C for several minutes, followed by the addition of the appropriate ligand (**L3** or **L4**) (1 eq.). The reaction was then stirred at 60 °C for further 16 h. After cooling to room temperature, all volatiles were removed under vacuum, the remaining crude was washed with Et_2_O (3 × 15 mL), dissolved in CH_2_Cl_2_ (100 mL) and washed with water (2 × 50 mL) and brine (2 × 50 mL). The organic extract was dried over Na_2_SO_4_, filtered, and all volatiles were evaporated to dryness under vacuum to yield the desired complexes as yellow crystalline solids.

**1**. Yield: 176 mg (58%). Anal. Found: C, 38.00; H, 2.95; N, 13.68. Calcd for C_13_H_12_BrMnN_4_O_3_ (407.10): C, 38.35; H, 2.97; N, 13.76. ^1^H NMR (400 MHz, DMSO-d_6_) δ: 9.11 (s, 1H, CH_py_), 8.11 (s, 2H, 2 × CH_py_), 7.54 (s, 1H, CH_py_), 4.73 (s, 2H, NCH_2_CH_3_) 4.54 (s, 3H, NCH_3_), 1.62 (s, 3H, NCH_2_CH_3_). ^13^C NMR (100 MHz, DMSO-d_6_) δ: 226.99 (CO), 222.51 (CO), 217.21 (CO), 188.65 (C_carbene_), 155.06, 149.26, 145.27, 138.89, 124.73, 121.21, 48.58 (NCH_2_CH_3_), 15.71 (NCH_2_CH_3_). IR (ν, cm^−1^): 2009 (vs), 1907 (vs), 1880 (vs).

**2**. Yield: 116 mg (46%). Anal. Found: C, 33.60; H, 3.36; N, 19.97. Calcd. for C_12_H_14_BrMnN_6_O_3_ (425.12): C, 33.90; H, 3.32; N, 19.77. ^1^H NMR (400 MHz, DMSO-d_6_) δ: 8.99 (s, 1H, CH_trz_), 4.68 (q, J = 7.31 Hz, 2H, NCH_2_CH_3_), 4.60 (q, J = 7.28 Hz, 2H, NCH_2_CH_3_), 4.35 (s, 3H, NCH_3_), 1.61 (t, J = 7.25 Hz, 3H, NCH_2_CH_3_), 1.55 (t, J = 7.33 Hz, 3H, NCH_2_CH_3_). ^13^C NMR (100 MHz, DMSO-d_6_) δ: 227.07 (CO), 222.95 (CO), 218.69 (CO), 185.83 (C_carbene_), 139.46, 139.18, 120.73, 48.59 (NCH_2_CH_3_), 46.63 (NCH_2_CH_3_), 37.63 (NCH_3_), 15.74 (NCH_2_CH_3_), 14.80 (NCH_2_CH_3_). IR (ν, cm^−1^): 2020 (vs), 1920 (vs).

#### 3.2.4. Preparation of Complex **3**

Solid [MnBr(CO)_5_] (1.1 eq.) and **L1** (1 eq.) were dissolved in dry CH_2_Cl_2_ (15 mL) and stirred at reflux during 16 h. After cooling to room temperature, the solvent was removed under vacuum, and the remaining solid was washed several times with ether to Yield **3** as a crystalline yellow solid.

**3**. Yield: 593 mg (83%). Anal. Found: C, 37.01; H, 2.68; N, 13.98. Calcd. for C_12_H_10_BrMnN_4_O_3_ (393.07): C, 36.67; H, 2.56; N, 14.25. ^1^H NMR (400 MHz, DMSO-d_6_) δ: 9.20 (s, 1H, CH_trz_), 9.09 (d, J = 5.39 Hz, 1H, CH_py_), 8.16 (t, J = 8.33 Hz, 1H, CH_py_), 7.61 (t, J = 5.39 Hz, 1H, CH_py_), 4.65 (q, J = 7.30 Hz, 2H, NCH_2_CH_3_), 1.57 (t, J = 7.30 Hz, 3H, NCH_2_CH_3_). ^13^C NMR (100 MHz, DMSO-d_6_) δ: 222.90 (CO), 221.67 (CO), 220.80 (CO), 153.36 (CH_py_), 148.68 (CH_py_), 146.47 (CH_py_), 139.29 (CH_py_), 125.12 (CH_trz_), 124.06(C_py_), 121.52 (C_trz_), 46.60 (NCH_2_CH_3_), 14.45 (NCH_2_CH_3_). IR (ν, cm^−1^): 2025 (vs), 1936 (vs), 1917 (vs).

### 3.3. Electrochemical Studies

For the electrochemical experiments, a potentiostat Autolab PGSTAT 12 AUT71019 controlled by NOVA 2.0 software was used. Cyclic voltammetry experiments were performed in a three-electrode one compartment electrochemical using glassy carbon (CHI Instruments, 3 mm diameter) and platinum wire were used as working and counter electrodes, respectively. An Ag wire immerged in the electrolyte solution was used as pseudo-reference and separated from the solution by a porous tip. Ferrocene was used as reference and all potentials are against Fc^+/0^_._ Tetrabutylammonium hexafluorophosphate (TBAPF_6_) as supporting electrolyte (recrystallised from hot ethanol). The working electrode was polished with alumina pastes of 1 and 0.05 μm diameter sizes and washed thoroughly with Milli-Q water and dried under a nitrogen flux. N_2_ or CO_2_ saturated CH_3_CN solutions with 1 mM of complexes were used.

Controlled potential electrolysis was performed in a two-compartment cell, where a platinum coil was immerged in a frit tube containing 0.5 M tetraethylammonium acetate (TEAAc) (99% Sigma Aldrich) + 0.2 M TBAPF_6_/MeCN electrolyte solution and separated from the cathodic compartment by a glass frit. In the catholyte compartment, 1 mM of complex was used with or without a proton source added, with a glassy carbon plate (Sigradur G) with 1.5 cm^2^. Silver wire as used as pseudo-reference immerged in a frit tube containing the same electrolyte solution (0.1 M TBAPF_6_/MeCN) and the redox potential of the Fc^+/0^ were checked to perform the experiments in the desired potential. N_2_ or CO_2_ was used to saturate the solutions. In the end of the experiment, gas chromatography with thermal conductivity detection (GC-TCD) was used to analyse the CO_2_ electroreduction products. An Agilent Technology (GC-TCD 7820A) controlled by OpenLAB CemStation edition software. A Carboxen^®^-1006 PLOT Capillary GC Column (L × I.D. 30 m × 0.53 mm, average thickness 30 μm) was used for H_2_, CO, CH_4_ and CO_2_ detection. Temperature was held at 230 °C for both the injector and detector. The carrier gas was Ar flowing at 3 mL·min^−1^ and injections were performed with gas tight syringes (500 μL) previously purged with CO_2_. The method used was based on keeping the oven at constant temperature 30 °C. Calibration curves were obtained for H_2_, CO and CH_4_ separately by injecting known volumes of pure gas in the electrochemical cell and taking a 500 μL aliquot to directly inject in the GC-TCD. The liquid phase was analysed by high performance liquid chromatography (HPLC), using a Waters chromatographer (Waters Chromatography, Milford, MA, USA), connected to a Waters 2998 Photodiode Array Detector set at 190 nm. Chromatographic separation was undertaken using an Aminex HPX-87H column (300 × 7.8 mm), 9 µm particle size (Bio-Rad, Hercules, CA, USA) and set at 60 °C. Elution was carried out isocratically, at a flow rate of 0.5 mL·min^−1^, with 0.005 N of H_2_SO_4_ and the volume inject was 50 μL. The formate retention time obtained was 16.2 min. Data acquisition was accomplished with the Empower 2 software (Waters Chromatography).

### 3.4. X-ray Diffraction Studies

Crystals suitable for single-crystal X-ray analysis of complexes **1** and **2** were selected, covered with Fomblin (polyfluoro ether oil) and mounted on a nylon loop. The data were collected at 110(2) K or at 296(2) K, for **1** and **2** respectively, on a Bruker D8 Venture diffractometer equipped with a Photon 100 CMOS detector and an Oxford Cryosystem Cooler, using graphite monochromated Mo-Kα radiation (λ = 0.71073 Å). The data were processed using the APEX3 suite software package, which includes integration and scaling (SAINT), absorption corrections [39] and space group determination (XPREP). Structure solution and refinement were performed using direct methods with the programs SHELXT 2018/2 and SHELXL (version 2018/3) [40,41] inbuilt in APEX, and WinGX-Version 2021.3 [42] software packages. The crystal of **1** showed poorer quality and diffracting power, giving rise to low quality data and, consequently, a low ratio of observed/unique reflections. This prevented the anisotropic refinement of all the carbon atoms. Moreover, **1** was refined as a 2-component inversion twin. Nevertheless, all characterization results are consistent with the remaining chemical characterization analysis and the model reported herein. In Complex **2**, the carbon atom C4 is disordered over two positions with 49 and 51%, respectively. Except for all carbon atoms in **1**, all non-hydrogen atoms were refined anisotropically. Hydrogen atoms were inserted in idealized positions and allowed to refine riding on the parent carbon atom. The molecular diagrams were drawn with ORTEP-3 (version 2020.1) [42], included in the software package. Crystal data for **1**: C_52_H_48_Br_4_Mn_4_N_16_O_12_, FW = 1628.42, monoclinic, space group *Pc* (no.7), *D_c_* = 1.718 g cm^−3^, Z = 2, *a* = 20.014(10), *b* = 8.430(4), *c* = 18.663(11) Å, α = 90, β = 90.009(17), γ = 90°, V = 3149(3) Å^3^, T = 110(2) K, Bruker D8 Venture diffractometer with Photon 100 CMOS area detector, λ (MoKα) = 0.71073 Å, µ = 3.392 mm^−1^. Of 57,690 reflections measured (*R*_int_ = 0.2828), 11,640 were unique. Refinement on F^2^ concluded with the values *R*_1_ = 0.0750 and *wR*_2_ = 0.1319 for 525 parameters and 3558 data with I > 2σI. Crystal data for **2**: C_12_H_14_BrMnN_6_O_3_, FW = 425.14, monoclinic, space group *P*2_1_/*c* (no.14), Dc = 1.649 g cm^−3^, Z = 4, *a* = 8.3118(4), *b* = 8.9797(4), *c* = 23.0161(11) Å, α = 90, β = 94.5180(10), γ = 90°, V = 1712.53(14) Å^3^, T = 296(2) K, Bruker D8 Venture diffractometer with Photon 100 CMOS area detector, λ (MoKα) = 0.71073 Å, µ = 3.126 mm^−1^. Of 34,815 reflections measured (*R*_int_ = 0.1077), 4254 were unique. Refinement on F^2^ concluded with the values *R*_1_ = 0.0516 and *wR*_2_ = 0.1338for 201 parameters and 3177 data with *I* > 2*σI*. The data were deposited in the CCDC under deposit numbers 2,112,120 for **1** and 2,112,121 for **2**.

## 4. Conclusions

In this work, the synthesis of a new family of manganese tricarbonyl complexes bearing triazolylidene/triazole/pyridine ligands of general formula *fac*-[Mn(L)(CO)_3_Br] [L = MIC^py (**1**), MIC^trz (**2**), trz^py (**3**)] have been reported. All complexes were characterized by IR and NMR spectroscopy, and by elemental analysis. Moreover, the crystal structures of **1** and **2** were determined by X-ray diffraction studies. The CO_2_-electrocatalytic reduction activity of complexes **1**–**3** and *fac*-[Mn(MIC^MIC)(CO)_3_Br] (**4**) have been investigated. For the first time, it has been explored the impact of the presence of a mesoionic triazolylidene ligand on the electrocatalytic activity of Mn tricarbonyl complexes. All compounds exhibited a catalytic current enhancement under an atmosphere of CO_2_. The best performance catalysts were complexes **3** and **4** that displayed a faradaic efficiency of 72% and 70%, respectively, for the selective production of CO. Interestingly, while the best efficiency for complex **3** was achieved when the experiments were performed in the presence of 1M of a protic source (water or TFE) at low overpotential (−1.86 V), complex **4** was performing best in neat acetonitrile at the higher potential of −2.14 V.

## Figures and Tables

**Table 1:** Selected NMR and IR data of Mn complexes **1**–**3**.

Complex	ν (CO) (cm^−1^)	^13^C NMR-C_carbene_ (ppm)
**1**	2009, 1907, 1880	188.65
**2**	2019, 1919	185.83
**3**	2025, 1963, 1917	--

**Table 2:** Summary of the CO_2_ electroreduction data from CV experiments for all Complexes **1**–**4** at *v* = 0.1 V s^−1^.

Complex	[H_2_O] (M) ^a^	E_cat/2_ (V vs. Fc^+/0^) ^b^	(i_cat_/i_p_)^2^
**1**	0.1	−2.35	2.62
**2**	0.1	−2.58	4.80
**3**	1.0/0.5	−1.86/−2.39 ^c^	30.1/22.1 ^c^
**4**	0	−2.14/−2.48 ^c^	29.81/39.1 ^c^

^a^ Optimized water concentration where the highest catalytic current was observed. ^b^ Calculated from the highest catalytic current observed. ^c^ Values taken for the two peaks observed under CO_2_ atmosphere as both show significant current increase when compared with N_2_ atmosphere.

**Table 3:** Summary of the controlled potential electrolysis data ^a^.

Entry	Complex	[acid] (M)	E_applied_ (V vs. Fc^+/0^) ^a^	FE_CO_ (%) ^b^
1	**3**	0	−1.89	56
2		0.5 (H_2_O)	−1.85	72
3		1.0 (H_2_O)	−1.89	62
4		1.0 (TFE)	−1.92	61
5	**4**	0	−2.08	69
6	**4**	0	−2.15	70

^a^ Measured in MeCN/0.2 M TBAPF_6_ with 1 mM catalyst and [acid] in the cathodic compartment. The anodic compartment with 0.2 M TBAPF_6_ and 0.5 M TEAAc in MeCN solutions. Electrolysis time: 1 h. ^b^ Formic acid was detected and quantified using HPLC with a FE < 8%.

## Data Availability

The data presented in this study are available from the authors.

## Supplementary Materials

The following are available online, Figure S1: ^1^H NMR spectrum (DMSO-d_6_, 400 MHz) of **L3**, Figure S2: ^13^C NMR spectrum (DMSO-d_6_, 100 MHz) of **L3**, Figure S3: ^1^H NMR spectrum (DMSO-d_6_, 400 MHz) of **L4**, Figure S4: ^13^C NMR spectrum (DMSO-d_6_, 100 MHz) of **L4**, Figure S5: ^1^H NMR spectrum (DMSO-d_6_, 400 MHz) of **1**, Figure S6: ^13^C NMR spectrum (DMSO-d_6_, 100 MHz) of **1**, Figure S7: IR spectrum of **1**, in KBr, Figure S8: ^1^H NMR spectrum (DMSO-d_6_, 400 MHz) of **2**, Figure S9: ^13^C NMR spectrum (DMSO-d_6_, 100 MHz) of **2**, Figure S10: IR spectrum of **2**, in KBr, Figure S11: ^1^H NMR spectrum (DMSO-d_6_, 400 MHz) of **3**, Figure S12: ^13^C NMR spectrum (DMSO-d_6_, 100 MHz) of **3**, Figure S13: IR spectrum of **3**, in KBr, Figure S14: Cyclic voltammograms of complexes **1**–**4** (1 mM) in MeCN/N_2_ saturated solutions using TBAPF_6_ as supporting electrolyte (0.1 M) at 0.1 V s^−1^. Glassy carbon (3 mm diameter) was used as working, platinum wire as counter and Ag wire pseudo-reference as electrodes, Figure S15: Cyclic voltammograms of complex **1** (1 mM) in MeCN/N_2_ or CO_2_ saturated solutions using TBAPF_6_ as supporting electrolyte (0.1 M) at 0.1 V s^−1^ in the presence of different [H_2_O]. Glassy carbon (3 mm diameter) was used as working, platinum wire as counter and Ag wire pseudo-reference as electrodes. Figure S16: Cyclic voltammograms of complex **2** (1 mM) in MeCN/N_2_ or CO_2_ saturated solutions using TBAPF_6_ as supporting electrolyte (0.1 M) at 0.1 V s^−1^ in the presence of different [H_2_O]. Glassy carbon (3 mm diameter) was used as working, platinum wire as counter and Ag wire pseudo-reference as electrodes, Figure S17: Plots of the activity (i_cat_/i_p_)^2^ vs. [H_2_O] measured at the highest current observed for complexes (a) **1**, **2** and (b) **3** and **4**. *v* = 0.1 V s^−1^, Figure S18: Cyclic voltammograms of complex **3** (1 mM) in MeCN/, N_2_ or CO_2_ saturated solutions using TBAPF_6_ as supporting electrolyte (0.1 M) at 0.1 V s^−1^ in the presence of different [TFE]. Glassy carbon (3 mm diameter) was used as working, platinum wire as counter and Ag wire pseudo-reference as electrodes, Figure S19: Plots of the activity (i_cat_/i_p_)^2^ vs. [TFE] measured at highest current observed for complex **3**. *v* = 0.1 V s^−1^, Figure S20: Cyclic voltammograms of complex **3** (1 mM) in MeCN, N_2_ saturated solution using TBAPF_6_ as supporting electrolyte (0.1 M) at 0.1 V s^−1^ in the presence of different [TFE]. Glassy carbon (3 mm diameter) was used as working, platinum wire as counter and Ag wire pseudo-reference as electrodes, Figure S21: (a) Current and charge (b) versus time during bulk electrolysis experiments with a CO_2_ saturated solution for **3** with or without a proton source, Figure S22: (a) Current and charge (b) versus time during bulk electrolysis experiments with a CO_2_ saturated solution for **5** without a proton source.

## Sample Availability

Samples of the compounds **1**–**4** are available from the authors.
