# Supplementary material for: Click-Derived Triazoles and Triazolylidenes of Manganese for Electrocatalytic Reduction of CO2
Source: Molecules. 2021 Oct 20;26(21):6325. doi: 10.3390/molecules26216325 (PMC8588546; doi:10.3390/molecules26216325)
Supplement: Supplementary file 1 [file molecules-26-06325-s001.zip › molecules-1422212-supplementary.pdf]

molecules

Supporting Information

Click-derived Triazoles and Triazolylidenes of Manganese for Electrocatalytic Reduction of CO_2

Sofia Friães [1], Sara Realista [1], Clara S. B. Gomes [2,3,4], Paulo N. Martinho [5,6] and Beatriz Royo [1,*]

1 ITQB NOVA, Instituto de Tecnologia Química e Biológica António Xavier, Av. da República, 2780-157 Oeiras, Portugal; sofiafriaes@itqb.unl.pt (S.F.); sara.realista@itqb.unl.pt (S.R.)
2 LAQV-REQUIMTE, Department of Chemistry, Campus de Caparica, NOVA School of Science and Technology, NOVA University Lisbon, 2829-516 Caparica, Portugal; clara.gomes@fct.unl.pt
3 Associated Laboratory i4HB-Institute for Health and Bioeconomy, School of Science and Technology, NOVA University Lisbon, 2829-516 Caparica, Portugal
4 UCIBIO-Applied Molecular Biosciences Unit, Department of Chemistry, School of Science and Technology, NOVA University Lisbon, 2829-516 Caparica, Portugal
5 Biosystems and Integrative Sciences Institute (BioISI), Faculdade de Ciências, Campo Grande, Universidade de Lisboa, 1749-016 Lisboa, Portugal; pnmartinho@fc.ul.pt
6 Centro de Química Estrutural, Campo Grande, Faculdade de Ciências Universidade de Lisboa, 1749-016 Lisboa, Portugal
* Correspondence: broyo@itqb.unl.pt
† Dedicated to Prof. Christian Bruneau for his outstanding contribution to catalysis.

Contents:

Characterization of ligands **L3** and **L4**	**S1**
Characterization of complexes **1-3**	**S4**
Cyclic voltammetry studies	**S9**
Bulk electrolysis	**S12**

1. Caracterization of ligands **L3** and **L4**

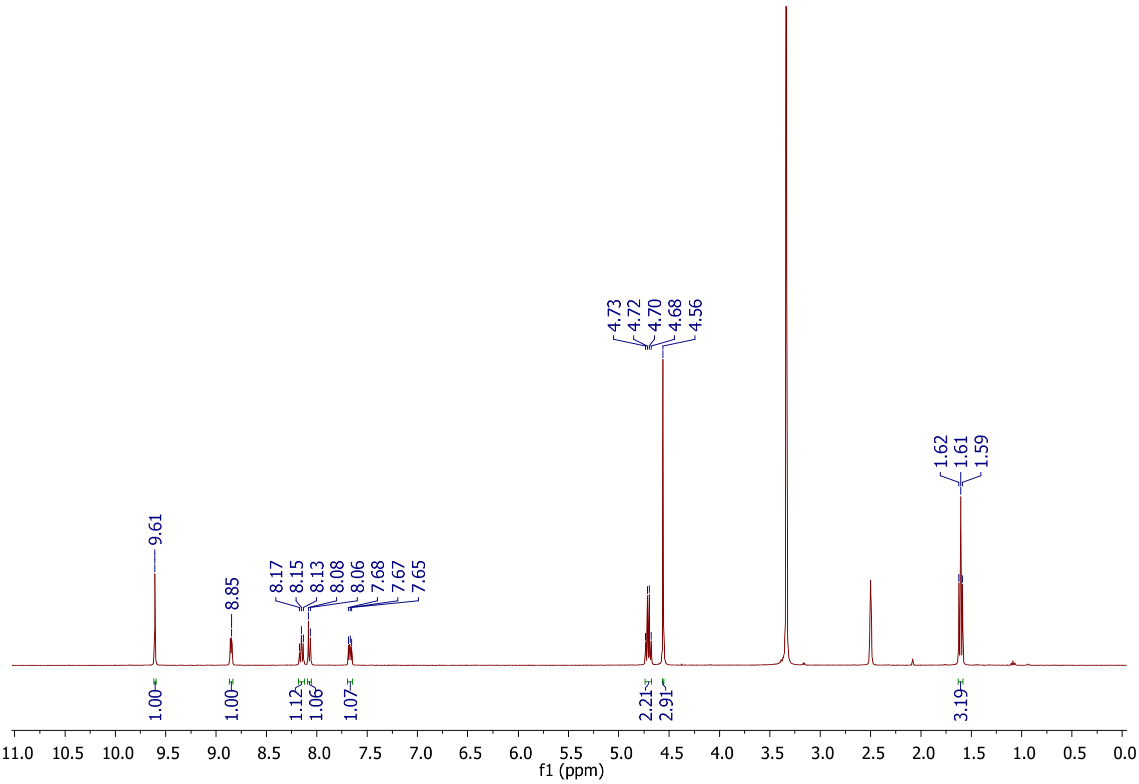

Figure S1. ^{1}H RMN spectrum (DMSO-d6, 400 MHz) of **L3**.

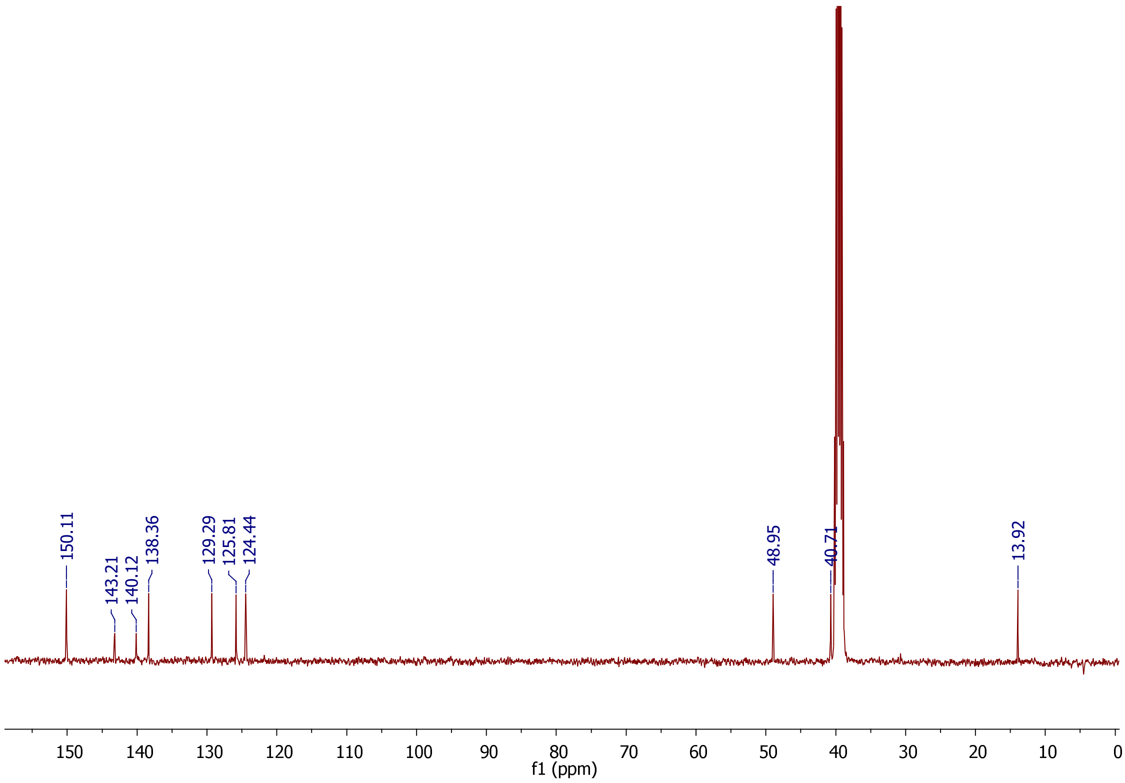

Figure S2. ^{13}C RMN spectrum (DMSO-d_6, 100 MHz) of **L3**.

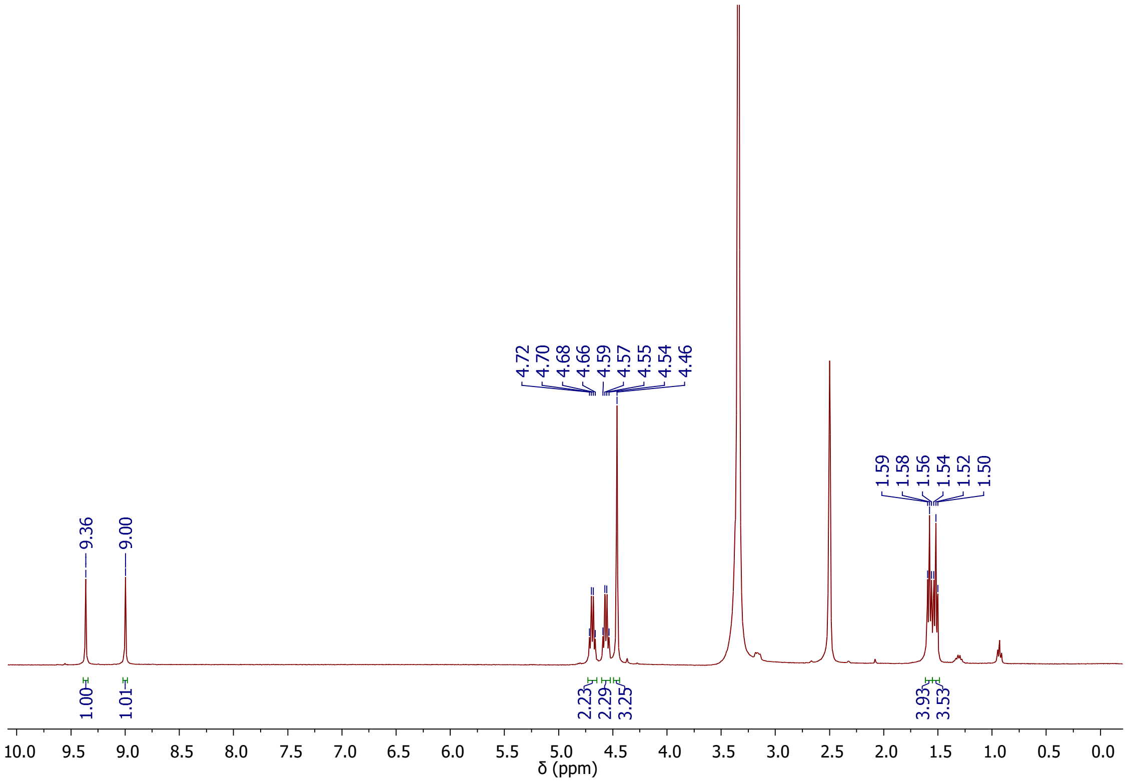

Figure S3. ^{1}H RMN spectrum (DMSO-d_6, 400 MHz) of **L4**.

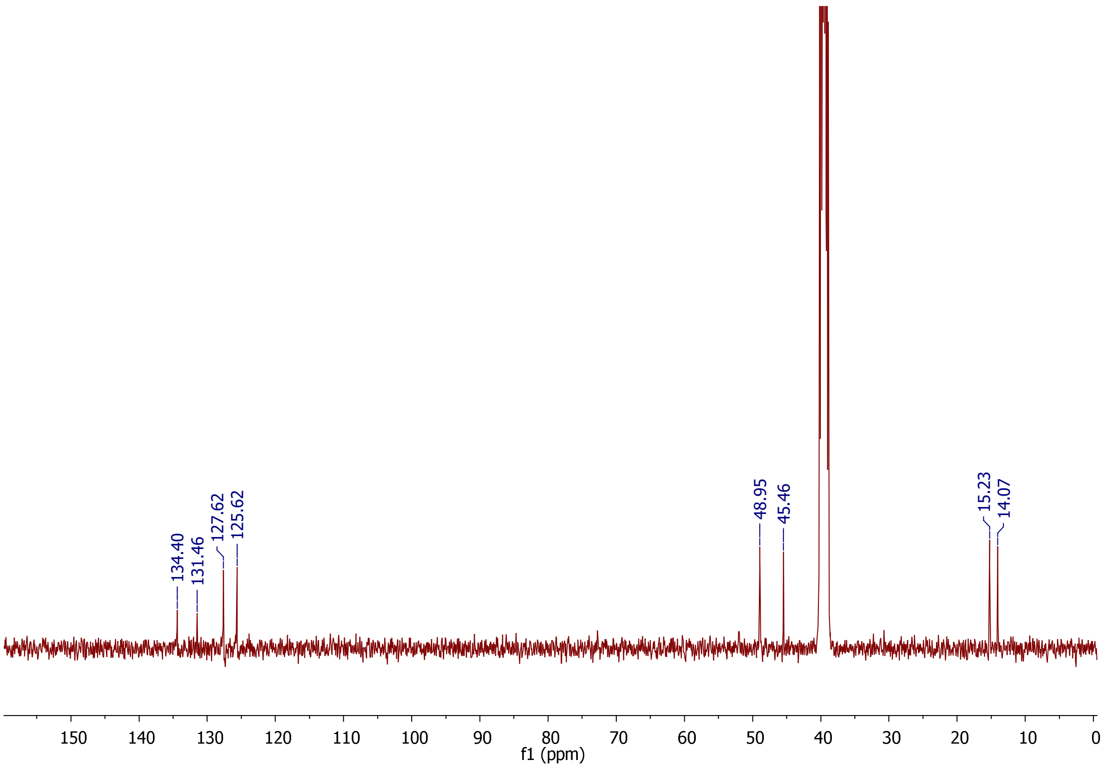

Figure S4. ^{13}C RMN spectrum (DMSO-d6, 100 MHz) of **L4**.

2. Characterization of complexes **1-3**.

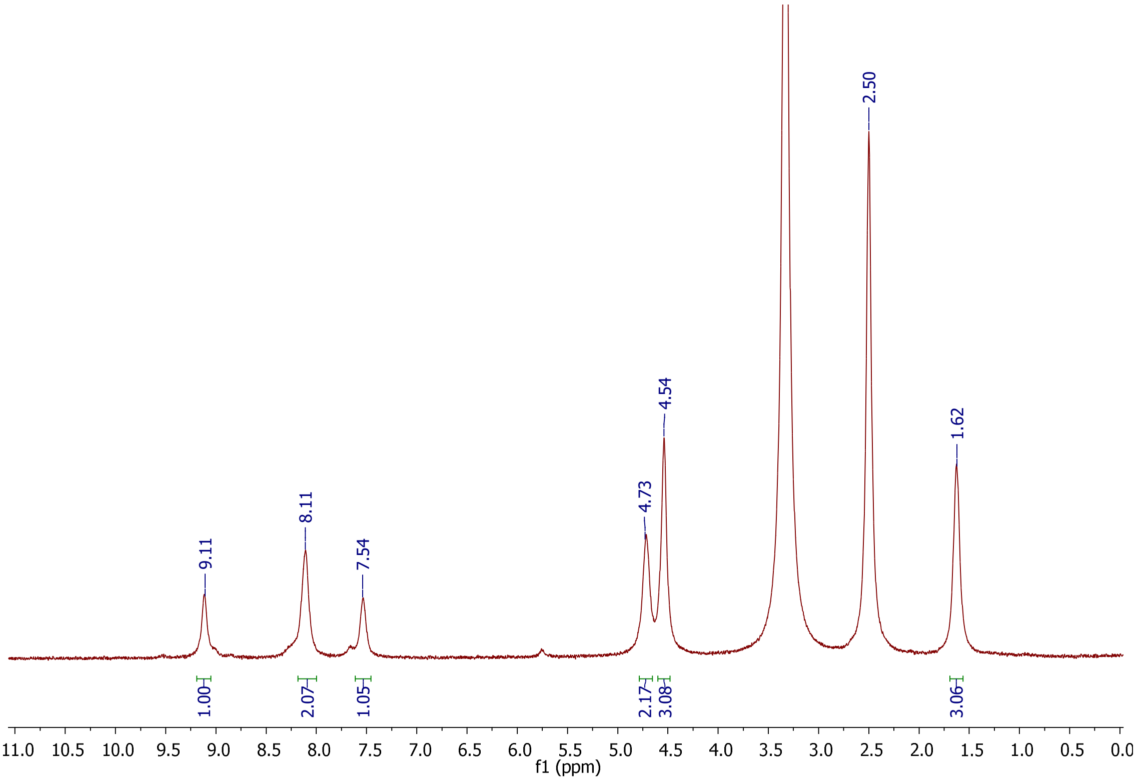

Figure S5. ^{1}H RMN spectrum (DMSO-d6, 400 MHz) of **1**.

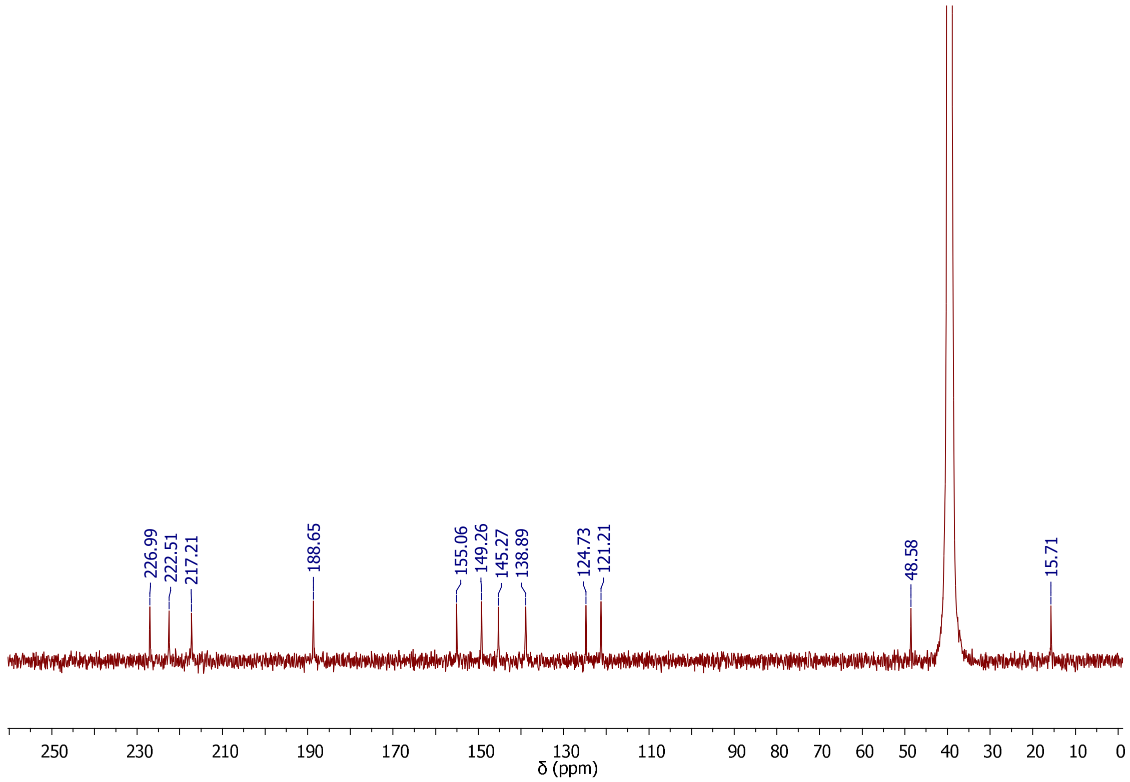

Figure S6. ^{13}C RMN spectrum (DMSO-d_6, 100 MHz) of **1**.

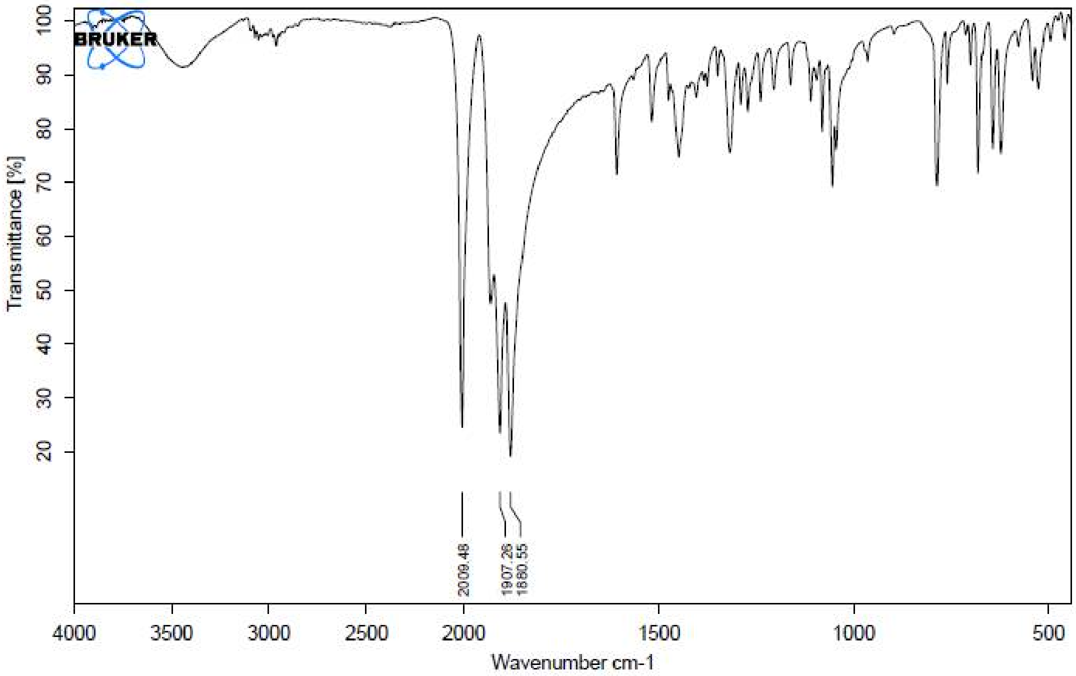

Figure S7. IR spectrum of **1**, in KBr.

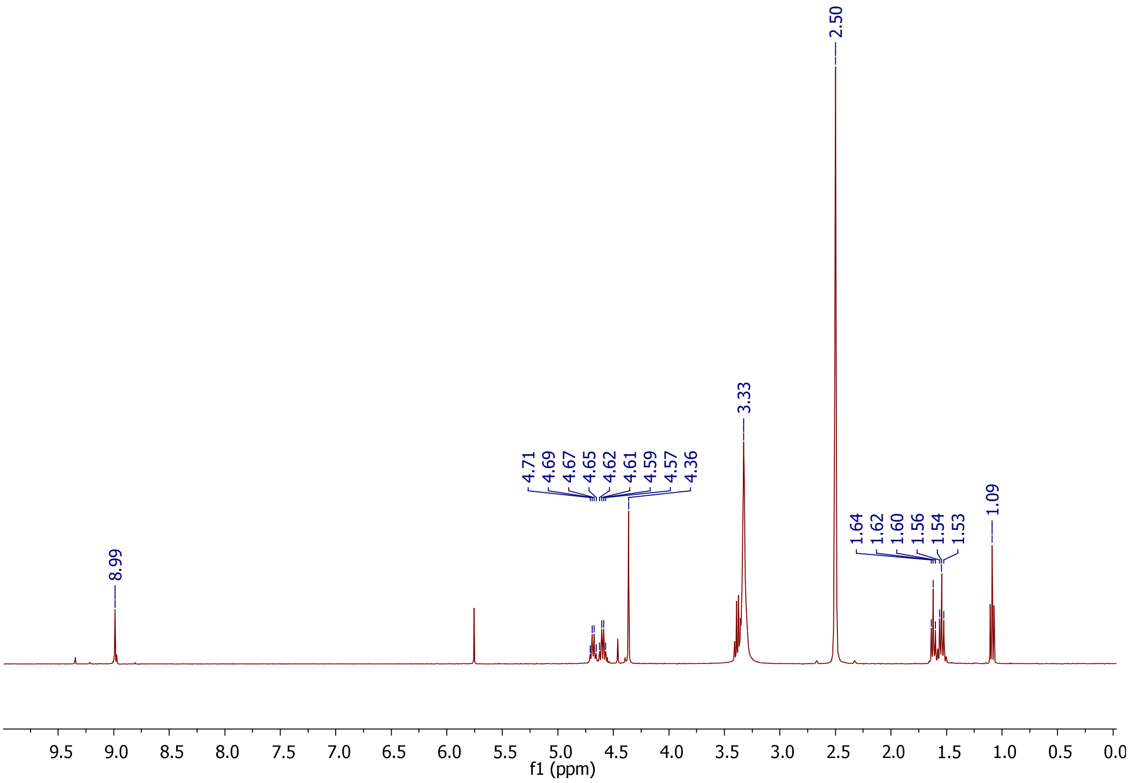

Figure S8. ^{1}H RMN spectrum (DMSO-d_6, 400 MHz) of **2**.

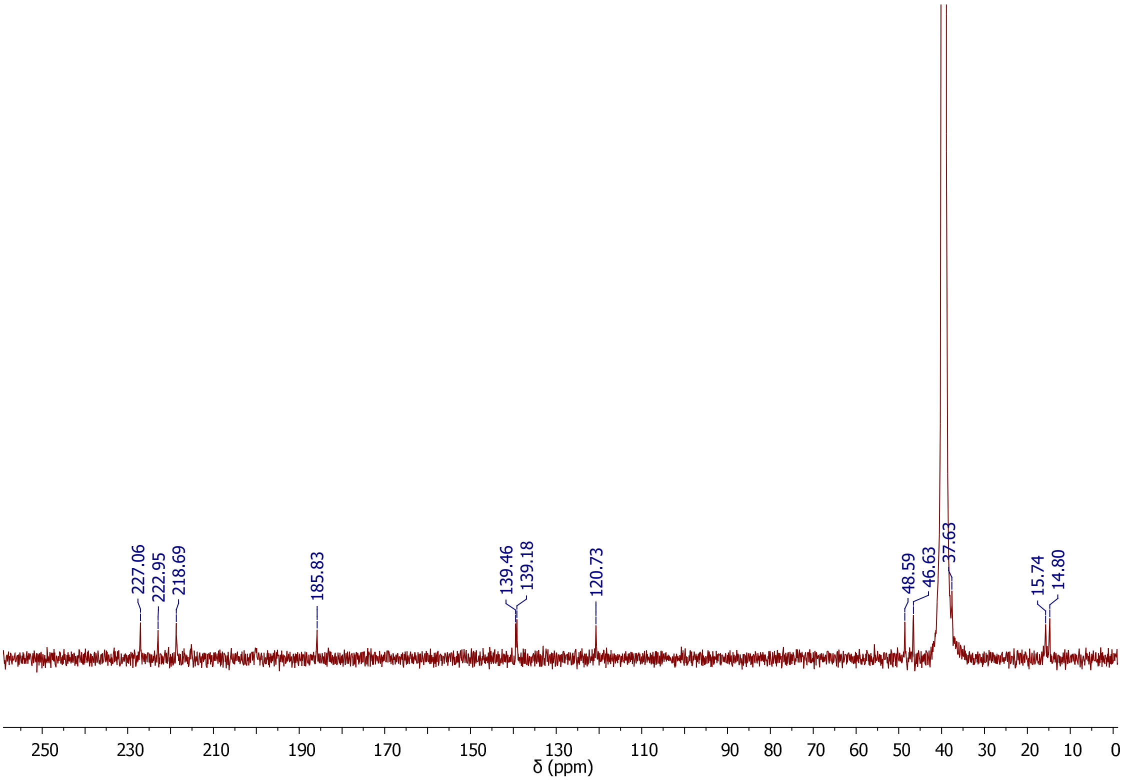

Figure S9. ^{13}C RMN spectrum (DMSO-d_6, 100 MHz) of **2**.

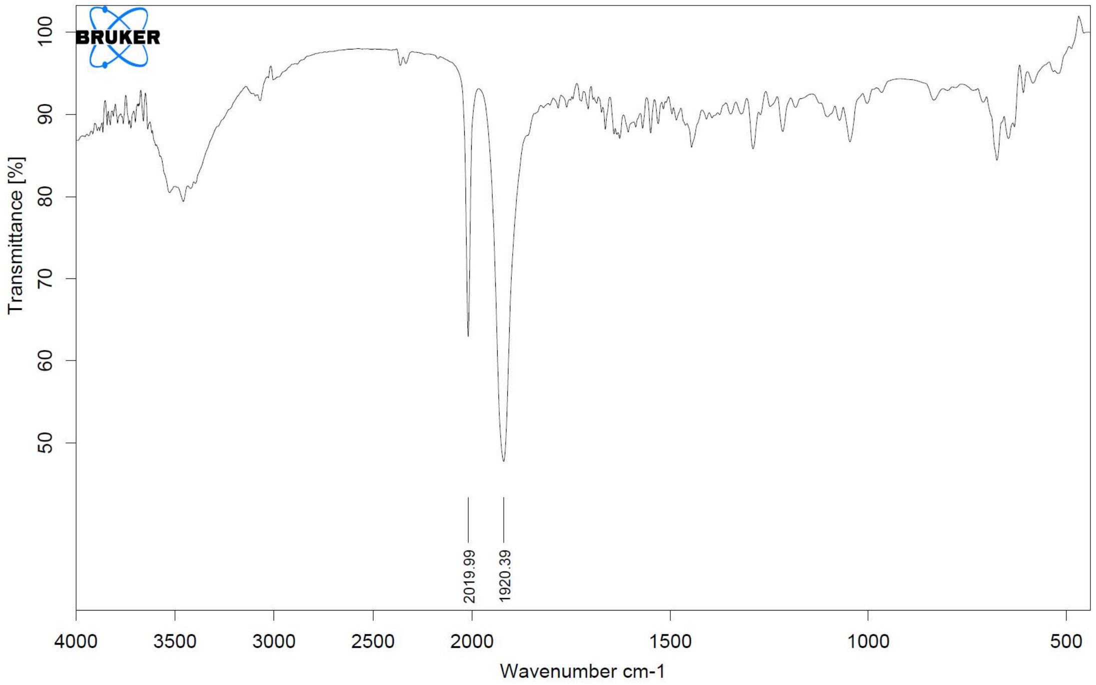

Figure S10. IR spectrum of **2**, in KBr.

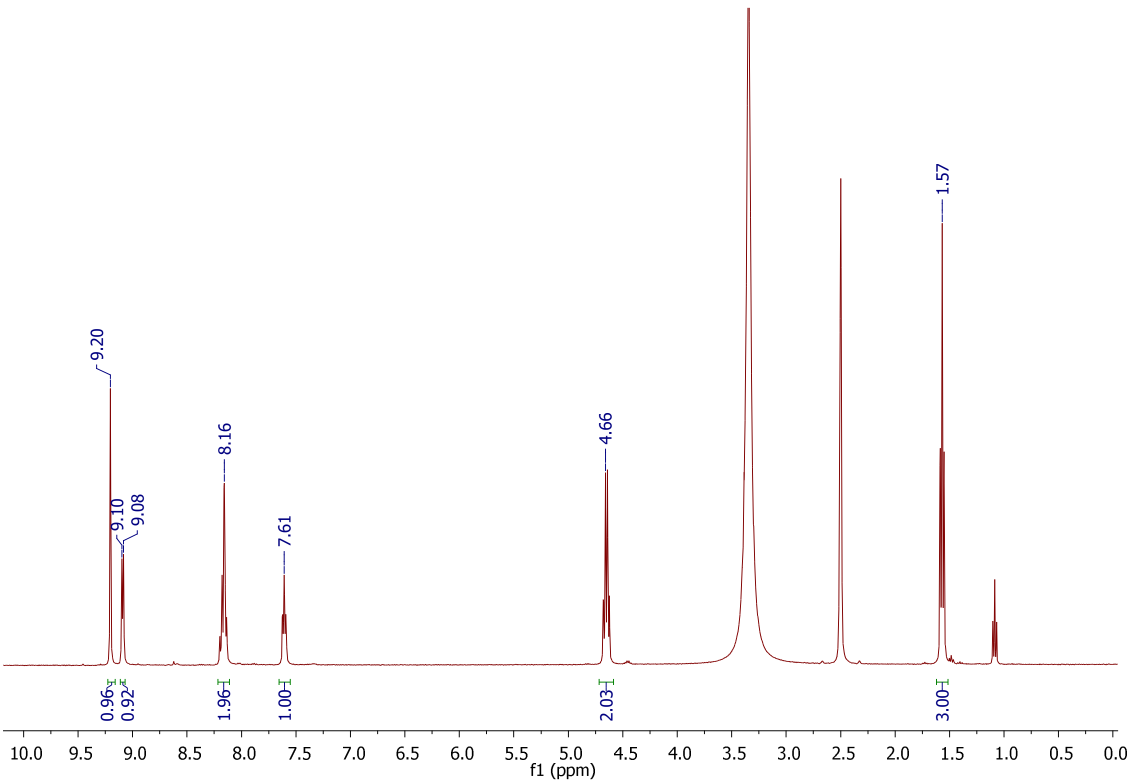

Figure S11. ^{1}H RMN spectrum (DMSO-d$_6$, 400 MHz) of **3**.

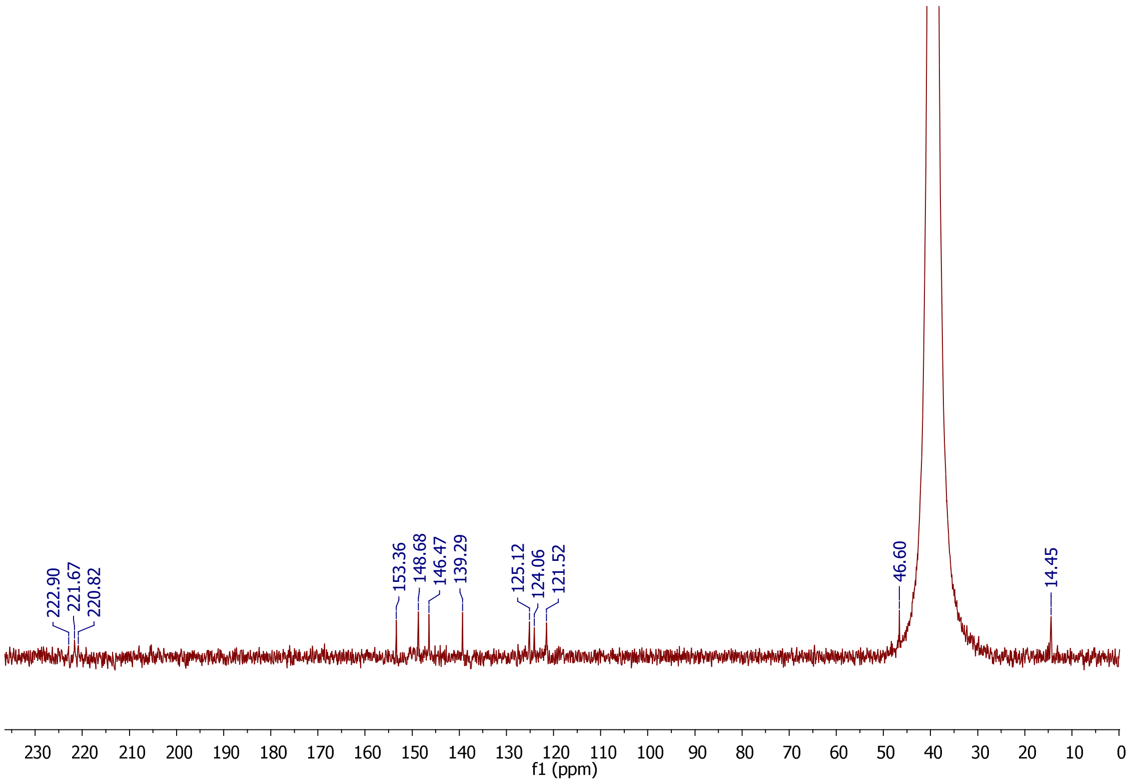

Figure S12. ^{13}C RMN spectrum (DMSO-d_6, 100 MHz) of **3**.

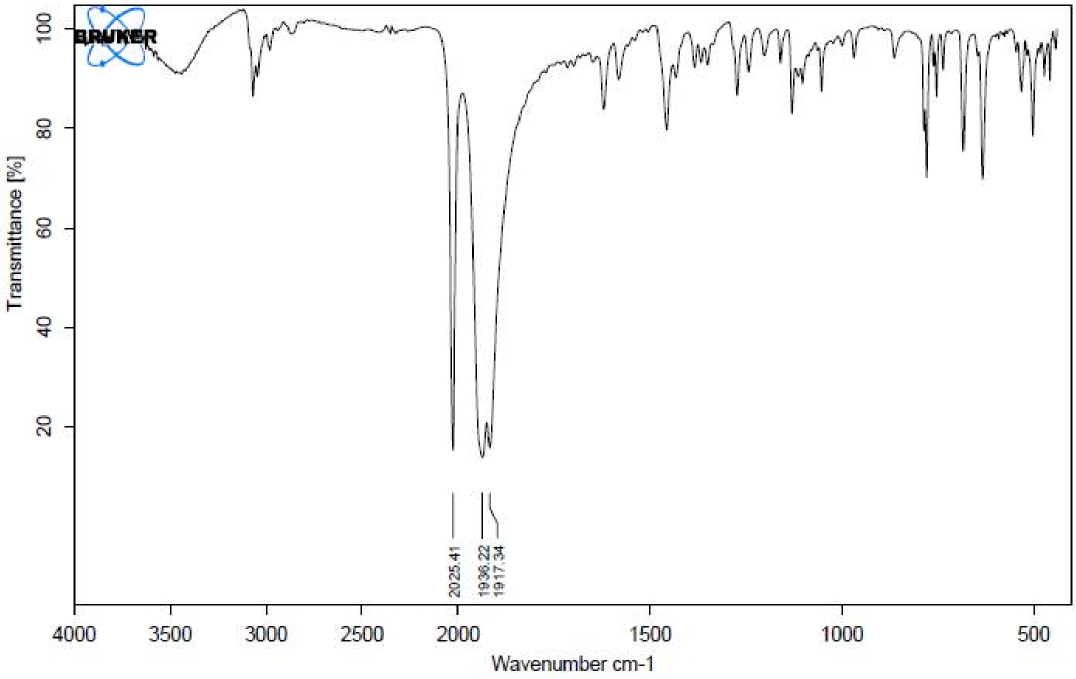

Figure S13. IR spectrum of **3**, in KBr.

3. Cyclic voltammetry

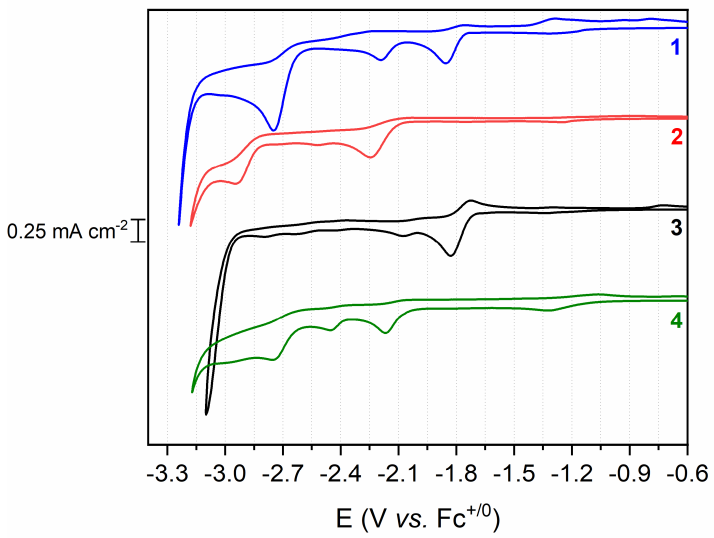

Figure S14. Cyclic voltammograms of complexes **1-4** (1 mM) in MeCN/, N_2 saturated solutions using $TBAPF_6$ as supporting electrolyte (0.1 M) at 0.1 V s^{-1}. Glassy carbon (3 mm diameter) was used as working, platinum wire as counter and Ag wire pseudo-reference as electrodes.

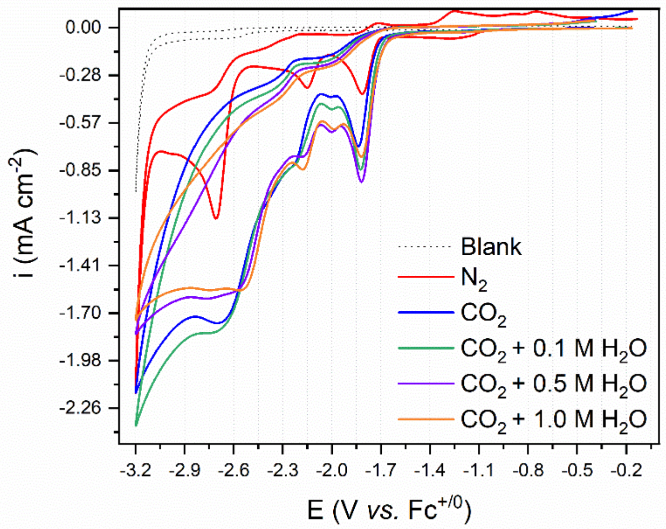

Figure S15. Cyclic voltammograms of complex **1** (1 mM) in MeCN/, N_2 or CO_2 saturated solutions using $TBAPF_6$ as supporting electrolyte (0.1 M) at 0.1 V s^{-1} in the presence of different [H_2O]. Glassy carbon (3 mm diameter) was used as working, platinum wire as counter and Ag wire pseudo-reference as electrodes.

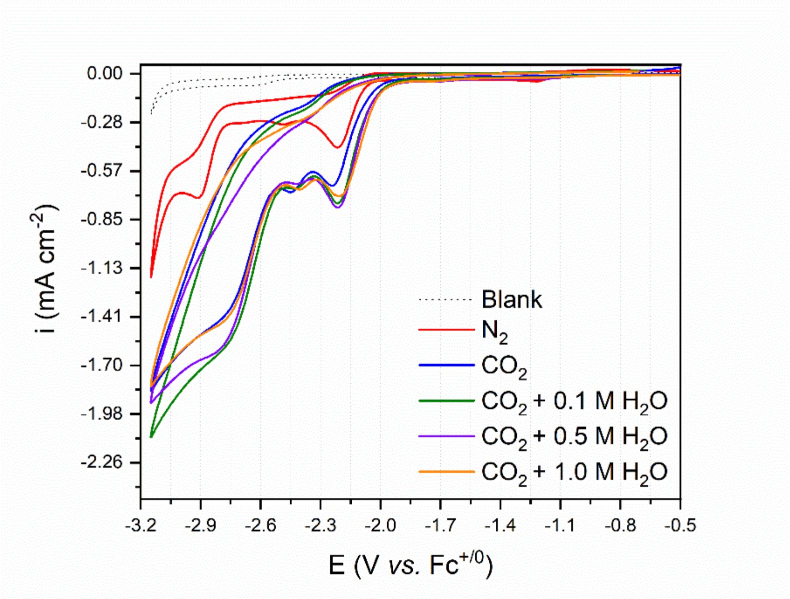

Figure S16. Cyclic voltammograms of complex **2** (1 mM) in MeCN/, N_2 or CO_2 saturated solutions using $TBAPF_6$ as supporting electrolyte (0.1 M) at 0.1 V s^{-1} in the presence of different [H_2O]. Glassy carbon (3 mm diameter) was used as working, platinum wire as counter and Ag wire pseudo-reference as electrodes.

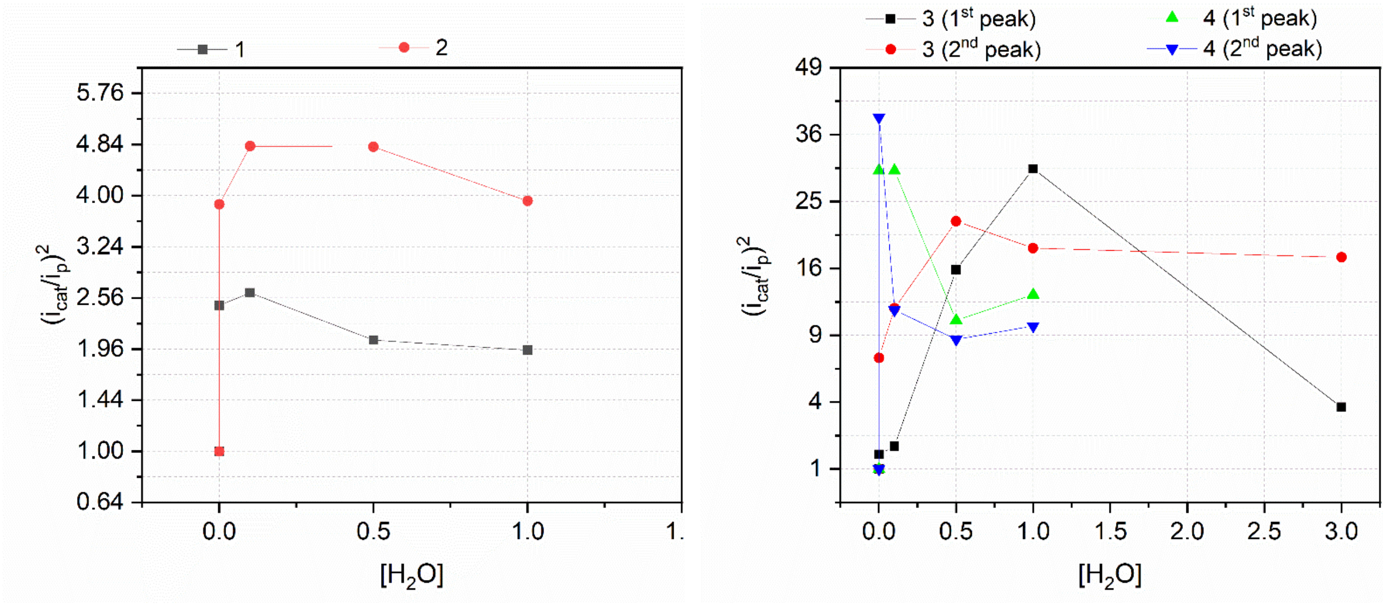

(**a**) (**b**)

Figure S17. Plots of the activity $(i_{cat}/i_p)^2$ *vs.* [H_2O] measured at the highest current observed for complexes a) **1**, **2** and b) **3** and **4**. v = 0.1 V s^{-1}.

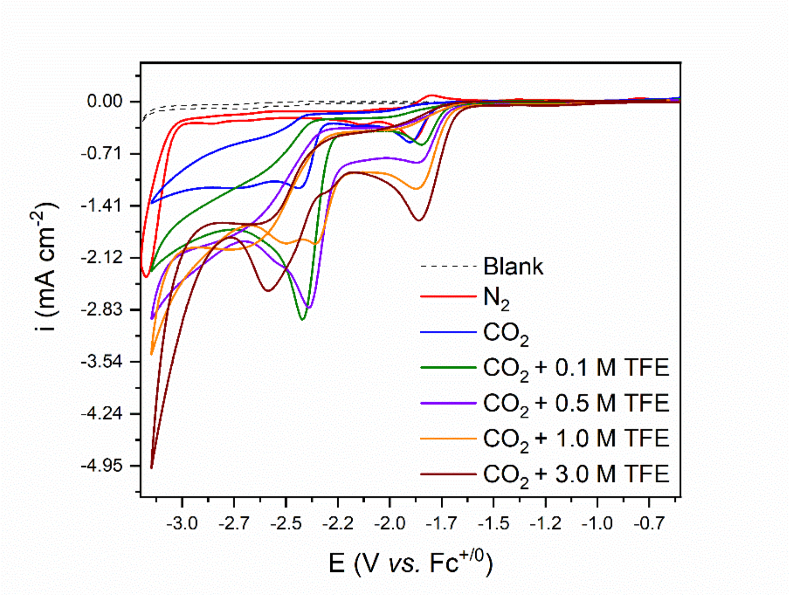

Figure S18. Cyclic voltammograms of complex **3** (1 mM) in MeCN/, N_2 or CO_2 saturated solutions using $TBAPF_6$ as supporting electrolyte (0.1 M) at 0.1 V s^{-1} in the presence of different [TFE]. Glassy carbon (3 mm diameter) was used as working, platinum wire as counter and Ag wire pseudo-reference as electrodes.

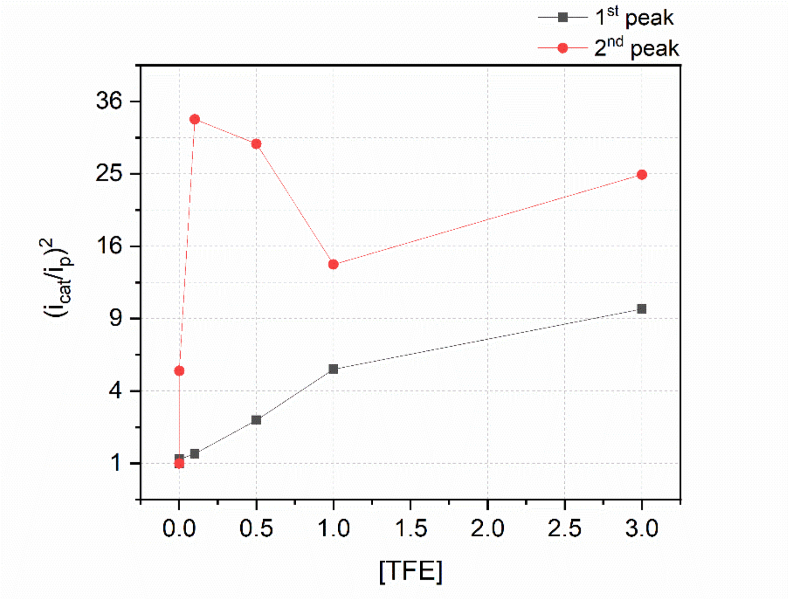

Figure S19. Plots of the activity $(i_{cat}/i_p)^2$ *vs.* [TFE] measured at highest current observed for complex **3**. v = 0.1 V s^{-1}.

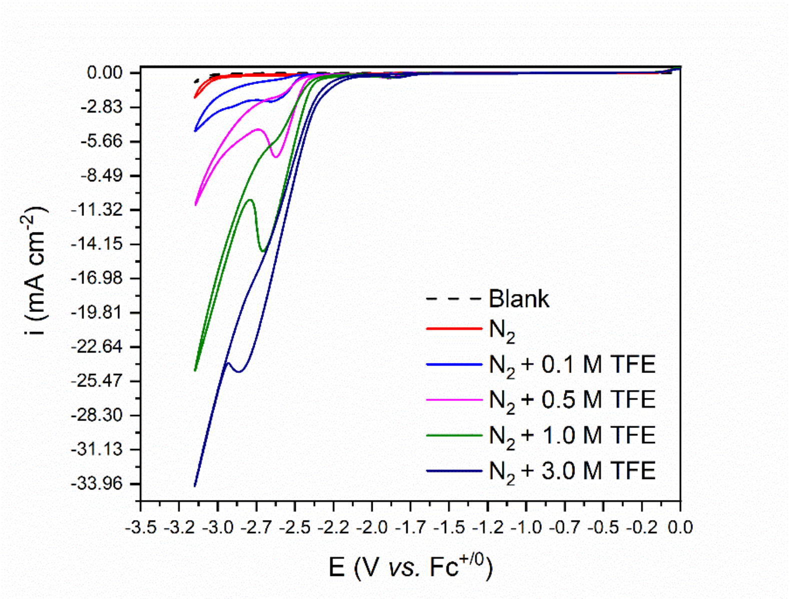

Figure S20. Cyclic voltammograms of complex **3** (1 mM) in MeCN, N_2 saturated solution using $TBAPF_6$ as supporting electrolyte (0.1 M) at 0.1 V s^{-1} in the presence of different [TFE]. Glassy carbon (3 mm diameter) was used as working, platinum wire as counter and Ag wire pseudo-reference as electrodes.

4. Bulk electrolysis

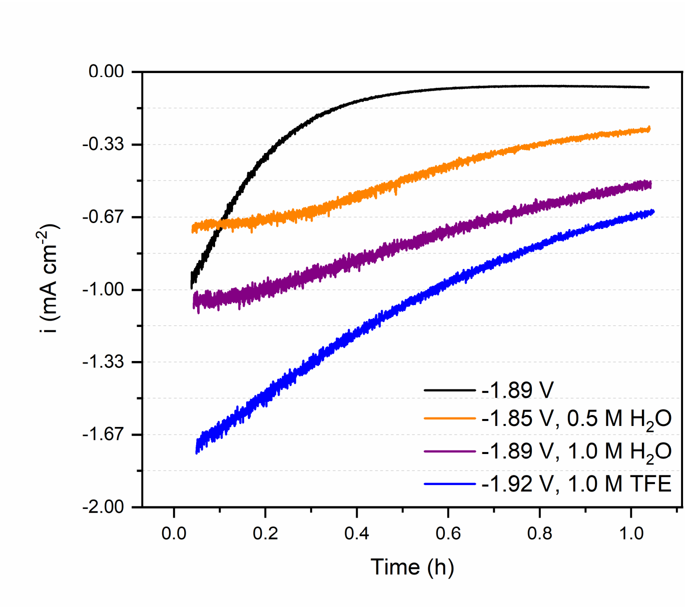

(**a**)

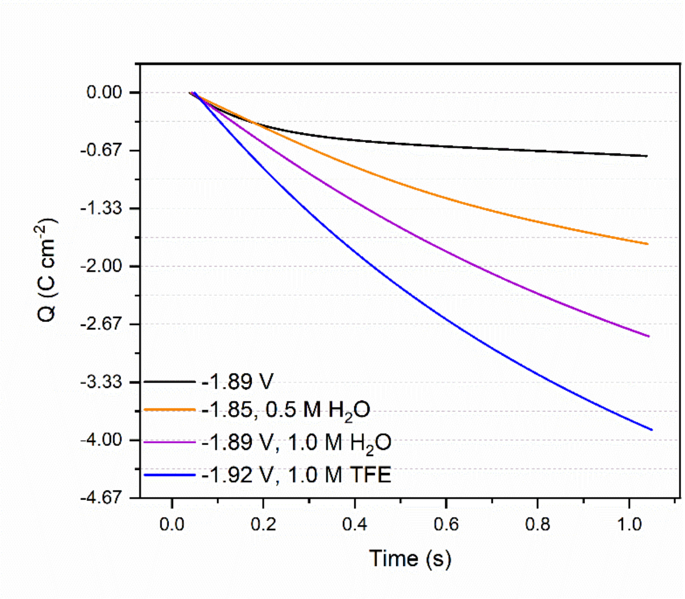

(**b**)

Figure S21. (a) Current and charge (b) versus time during bulk electrolysis experiments with a CO_2 saturated solution for **3** with or without a proton source.

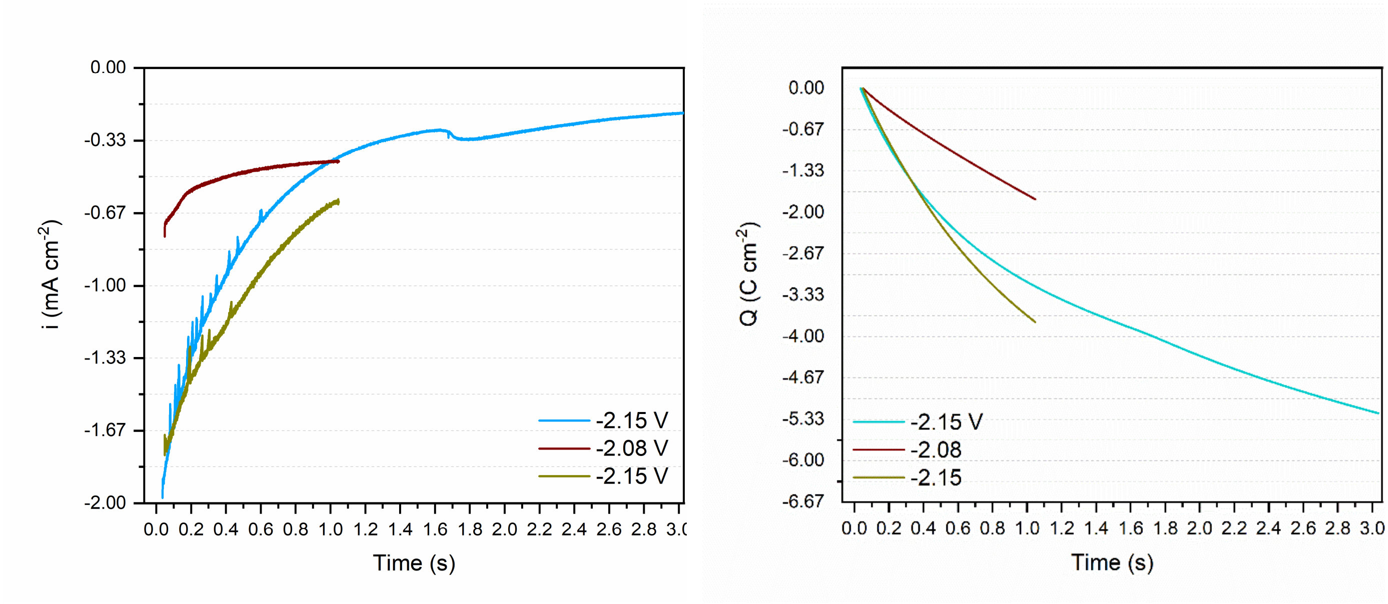

(**a**) (**b**)

Figure S22. (a) Current and charge (b) versus time during bulk electrolysis experiments with a CO_2 saturated solution for **5** without a proton source.
